# *In Vivo* Hematopoietic Stem Cell Gene Therapy for SARS-CoV2 Infection Using a Decoy Receptor

**DOI:** 10.1089/hum.2021.295

**Published:** 2022-04-19

**Authors:** Hongjie Wang, Chang Li, Adebimpe O. Obadan, Hannah Frizzell, Tien-Ying Hsiang, Sucheol Gil, Audrey Germond, Connie Fountain, Audrey Baldessari, Steve Roffler, Hans-Peter Kiem, Deborah H. Fuller, André Lieber

**Affiliations:** ^1^ Division of Medical Genetics, Department of Medicine, University of Washington, Seattle, Washington, USA.; ^2^ Department of Microbiology, University of Washington, Seattle, Washington, USA.; ^3^ Department of Immunology, University of Washington, Seattle, Washington, USA.; ^4^ Washington National Primate Research Center, Seattle, Washington, USA.; ^5^ Institute of Biomedical Sciences, Academia Sinica, Taipei, Taiwan.; ^6^ Stem and Gene Therapy Program, Fred Hutchinson Cancer Research Center, Seattle, Washington, USA.; ^7^ Department of Pathology, University of Washington, Seattle, Washington, USA.

**Keywords:** SARS-CoV2, *in vivo* HSC gene therapy, erythroid cells, decoy receptor

## Abstract

While SARS-CoV2 vaccines have shown an unprecedented success, the ongoing emergence of new variants and necessity to adjust vaccines justify the development of alternative prophylaxis and therapy approaches. Hematopoietic stem cell (HSC) gene therapy using a secreted CoV2 decoy receptor protein (sACE2-Ig) would involve a one-time intervention resulting in long-term protection against airway infection, viremia, and extrapulmonary symptoms. We recently developed a technically simple and portable *in vivo* hematopoietic HSC transduction approach that involves HSC mobilization from the bone marrow into the peripheral blood stream and the intravenous injection of an integrating, helper-dependent adenovirus (HDAd5/35^++^) vector system. Considering the abundance of erythrocytes, in this study, we directed sACE2-Ig expression to erythroid cells using strong β-globin transcriptional regulatory elements. We performed *in vivo* HSC transduction of CD46-transgenic mice with an HDAd-sACE2-Ig vector. Serum sACE2-Ig levels reached 500–1,300 ng/mL after *in vivo* selection. At 22 weeks, we used genetically modified HSCs from these mice to substitute the hematopoietic system in human ACE2-transgenic mice, thus creating a model that is susceptible to SARS-CoV2 infection. Upon challenge with a lethal dose of CoV2 (WA-1), sACE2-Ig expressed from erythroid cells of test mice diminishes infection sequelae. Treated mice lost significantly less weight, had less viremia, and displayed reduced cytokine production and lung pathology. The second objective of this study was to assess the safety of *in vivo* HSC transduction and long-term sACE2-Ig expression in a rhesus macaque. With appropriate cytokine prophylaxis, intravenous injection of HDAd-sACE2-Ig into the mobilized animal was well tolerated. *In vivo* transduced HSCs preferentially localized to and survived in the spleen. sACE2-Ig expressed from erythroid cells did not affect erythropoiesis and the function of erythrocytes. While these pilot studies are promising, the antiviral efficacy of the approach has to be improved, for example, by using of decoy receptors with enhanced neutralizing capacity and/or expression of multiple antiviral effector proteins.

## Introduction

During the past 5 years, we developed a new *in vivo* hematopoietic stem cell (HSC) gene therapy approach. It involves the mobilization of HSCs from the bone marrow by granulocyte colony-stimulating factor (G-CSF) and the short-acting CXCR4 antagonist Plerixafor/AMD3100. While mobilized HSCs circulate at high numbers in the periphery, HDAd5/35^++^ vectors are injected intravenously.^1^ Mobilization of HSCs is critical for *in vivo* transduction because in the bone marrow, they are surrounded by extracellular stroma proteins,^2^ and are not accessible to gene transfer vectors.^3^

HDAd5/35^++^ vectors are easy to manufacture at high yields, can carry a payload of 35 kb, and efficiently transduce primitive, quiescent HSCs through CD46.^3^ The vectors' affinity to CD46 has been increased^4^ to allow for *in vivo* HSC transduction without significant vector uptake by hepatocytes.^5,6^

Random integration of HDAd5/35^++^ vectors is mediated by an activity-enhanced *Sleeping Beauty* transposase (SB100x).^7^ To expand transduced HSCs, we currently use an *in vivo* selection mechanism based on a mutant O^6^-methylguanine-DNA methyltransferase (*mgmt^P140K^*) gene that confers resistance to O^6^-BG/BCNU (O^6^-Benzylguanine/Carmustine).^8–10^ After *in vivo* transduction and selection with three low doses of O^6^-BG/BCNU administered intraperitoneally with an interval of 2 weeks, transgene marking in peripheral blood mononuclear cells (PBMCs) is usually increased to >90%.^8^ Our approach resulted in phenotypic correction in mouse disease models of thalassemia intermedia,^11^ sickle cell disease, and murine hemophilia A,^12^ and in the reversion of spontaneous cancer.^13^ Recently, we also demonstrated its safety and efficacy in rhesus macaques.^14^

We hypothesized that the abundance of erythroid cells can be harnessed for high-level production of therapeutic proteins (84% of the cells in the human body are red blood cells [20–30 trillion] and 2.5 × 10^6^ erythrocytes are made per second). We demonstrated that our approach allows for stable supraphysiological plasma concentrations of a bioengineered human factor VIII, termed ET3, which was expressed from an erythroid-specific β-globin locus control region (LCR).^12^ Our study also suggested the ET3 was secreted from erythroid cells in the bone marrow or released from circulating erythrocytes during senescence. Despite high-level ET3 production from erythroid cells, no effect on hematopoiesis was observed.

The SARS-CoV2 spike (S) protein binds to ACE2 to infect cells.^15^ The main entry route for the virus is through the respiratory system and the first target of infection are airway epithelial cells resulting in lung inflammation and damage. However, even mild SARS-CoV2 infections can trigger a wide range of extrapulmonary symptoms, including loss of smell and taste, vomiting and diarrhea, and neurologic impairments. More critical, long-lasting sequelae of infection can involve the heart, kidney, brain, and GI-tract. It remains unclear which aspects of disease are the result of disseminated virus infection of multiple tissues versus dysregulation of signaling pathways, including cytokine storms.

The widespread tissue distribution of ACE2^16^ and the presence of virus in other tissues^17,18^ argue for the first one. ACE2 is not only expressed on lung alveolar epithelial cells but also on enterocytes of the small intestine, in arterial and venous endothelial cells, and arterial smooth muscle cells in all organs studied, testis, heart, kidney, liver, brain, and thyroid gland.^19–21^ Furthermore, viremia after lytic infection of lung epithelium is documented by reports that measured both infectious particles and/or viral RNA in blood, whereby the virus load in blood correlated strongly with disease severity.^22^

To disrupt the virus replication and pathological effects, monoclonal antibodies directed against the S-protein are being used.^23,24^ Another group of therapeutics are virus decoy receptors. The development of recombinant ACE2-Fc fusion proteins (hACE2-Fc or hACE2-Ig) started early in the pandemic.^25–30^ Unlike S protein neutralizing antibodies, hACE2-Fc treatment did not induce the development of escape mutants. The latter is based on the virology dogma that it is highly unlikely that the virus changes its primary entry receptor.

Pharmacokinetic data indicated that hACE2-Fc has a relative long half-life *in vivo* compared to soluble ACE2. This field of research has then progressed rapidly. New decoy receptors include a trimeric hACE2-Fc variant^30^ and a high-affinity tetravalent format ACE2-Fc-TD.^27^ This variant also had a human IgG domain completely devoid of binding to Fcγ receptors (FcγRs) and complement protein C1q, thereby reducing the risk of antibody-dependent enhancement of infection.^28^ Ongoing work utilizes bat ACE2 orthologs.^29^

Recombinant ACE2-Fc proteins cannot be used prophylactically long term. The need for repeat administration of recombinant aACE2-Fc in COVID-19 therapy is clearly a limitation. The direct expression of the protein from the host's tissues could address this issue. Jim Wilson's groups tested an rAAV vector expressing an affinity-enhanced ACE2-Fc and demonstrated that the expression of this decoy receptor in the proximal airway significantly diminished clinical and pathologic consequences of SARS-CoV2 challenge in a mouse model.^31^ They also showed that therapeutic levels of sACE2-Fc can be achieved in nonhuman primates after intranasal delivery.

We decided to test the *in vivo* HSC gene therapy approach for COVID-19 prophylaxis and therapy for the following reasons: our approach is a one-time intervention with intravenous injections and is therefore portable and has the potential for widespread application. It results in continuous release of sACE2-Ig from erythroid cells into the blood stream. High plasma sACE2-Ig levels could block viremia and extrapulmonary symptoms. Because sACE2-Ig is expressed continuously, it could be used prophylactically in individuals who cannot be vaccinated (*e.g.,* hypersensitive patients) or lack a functional immune system (*e.g.,* HIV/AIDS patients).

The major goals of this study were (i) to evaluate the therapeutic effect of *in vivo* HSC gene therapy with an HDAd-sACE2-Ig vector in mice challenged with a deadly dose of SARS-CoV2 and (ii) to gather first safety data of the approach in nonhuman primates (NHPs).

## Materials and Methods

Reagents, generation of HDAd5/35^++^ vectors, generation of sACE2-Ig protein, Western blotting, and vector copy number (VCN) measurement are described in Supplementary Methods.

### Pseudovirus neutralization assay

HEK293T cells stably expressing human ACE2 (293/ACE2, cat# SL221) and firefly luciferase lentiviral particles pseudotyped with truncated SARS-COV-2 spike glycoprotein (cat# SP001) were purchased from GeneCopeia (Rockville, MD). Purified protein or mouse serum containing sACE2-Ig was incubated with pseudotyped virus for 1 h before infection of 293/ACE2 cells, luciferase activities were measured by Luciferase Assay Systems (Promega) 60 h after infection, and the percent of neutralization was calculated.

### sACE2-Ig enzyme-linked immunosorbent assay

Recombinant SARS-CoV2 S1 protein (cat# Z03501; Genscript) was diluted to 1.5 μg/mL in phosphate-buffered saline (PBS) and immobilized on 96-well enzyme-linked immunosorbent assay (ELISA) plates overnight at 4°C. After blocking with StartingBlock blocking buffer (Thermo Scientific), MO7e culture medium or diluted serum samples were added to the wells. An anti-influenza hemagglutinin (HA)-tag antibody conjugated with horseradish peroxidase (HRP; Cell Signaling) was used to detect binding.

### SARS-CoV2 plaque assay

Briefly, 0.05–0.15 g freshly collected lung tissue was weighted and homogenized within 1 mL PBS in a Precellys Tissue Homogenizer using ceramic beads; debris were removed by centrifugation. VeroE6/TMPRSS2 cells were seeded in tissue culture-treated 12-well plates at 2.5 × 10^5^ per well in Dulbecco's modified Eagle's medium (DMEM; high glucose, l-glutamine, sodium pyruvate, HEPES, 1 × antibiotic-antimycotic, 1% heat-inactivated fetal bovine serum, and geneticin). The next day, media were removed and samples were serially diluted and added to cells in duplicate. Plates were incubated for 1 h at 37°C, rocking every 15 min.

Overlay media (0.2% agarose in DMEM) were added to each well and plates were incubated for 2 days at 37°C. Cells were fixed with 10% formaldehyde in PBS and incubated at room temperature for 30 min. Overlays were removed and cells were stained with 0.2% crystal violet in 70% EtOH. Plates were washed with tap water and allowed to dry. Plaques were enumerated and the average number of plaques per tissue sample was calculated.

### Real-time quantitative reverse transcription PCR quantification of SARS-CoV2 viral load

Total RNA from lung was extracted by homogenizing 0.05–0.15 g lung tissue in 1 mL Trizol, followed by using RNeasy Mini Kit (Qiagen). Total RNA from total blood cells was extracted by using RNeasy Mini Kit (Qiagen). Total RNA from 50 to 150 μL serum was extracted by QIAamp Viral RNA Mini Kit (Qiagen) by following manufacturer's instruction. To measure the viral RNA levels, TaqPath 1-Step RT-qPCR Master Mix (cat# A15299; Thermo Fisher) was used to set up the real-time quantitative reverse transcription PCR (qRT-PCR) reaction (20 μL/reaction with duplicate), maximum of 400 ng RNA sample per reaction was loaded; CDC primers and a FAM-labeled probe targeting the N2 amplicon of N gene (2019-nCoV RUO Kit, cat# 10006713; IDT-DNA) of SARS-CoV2 (accession MN908947) were used.

Amplification was performed in a LightCycler 480 real-time PCR system (Roche) with the following protocol: 50°C for 15 min and 95°C for 2 min, followed by 40 cycles of 95°C for 3 s and 60°C for 30 s. Viral genome copies were determined by comparison to a standard curve generated by using the plasmid containing SARS-CoV2 N gene (cat# 10006625; IDT-DNA). For samples that needed to be normalized for RNA loading, the PCR reaction was multiplexed with predesigned primers and Cy5-labeled probe specific to mouse beta actin gene (Assay ID. Mm.PT.39a.22214843.g, IDT-DNA), and compared with a standard curve generated by using known amount of total RNA extracted from mouse bone marrow.

### qRT-PCR of cytokine mRNA

qRT-PCR were performed as described above with predesigned primers and FAM-labeled probes specific to mouse interleukin (IL)-6 (Assay ID. Mm.PT.58.10005566, IDT-DNA). The PCR reactions were multiplexed with predesigned primers and Cy5-labeled probe specific to mouse beta actin gene (Assay ID. Mm.PT.39a.22214843.g, IDT-DNA) for normalization.

### IL-6 ELISA

The ELISA MAX Deluxe set Mouse IL-6 (Biolegend) was used according to the manufacturer's instructions.

### Animals

All experiments involving animals were conducted in accordance with the institutional guidelines set forth by the University of Washington. The University of Washington is an Association for the Assessment and Accreditation of Laboratory Animal Care International (AALAC)—accredited research institution and all live animal work conducted at this university is in accordance with the Office of Laboratory Animal Welfare (OLAW) Public Health Assurance (PHS) policy, USDA Animal Welfare Act and Regulations, the Guide for the Care and Use of Laboratory Animals, and the University of Washington's Institutional Animal Care and Use Committee (IACUC) policies.

The studies were approved by the University of Washington IACUC (Protocol No. 3108-01 for mice and 3108-04 for NHPs). Mice were housed in specific pathogen-free facilities. NHP studies were performed by the Washington National Primate Research Center (WaNPRC). SARS-CoV2 challenge studies were performed under BSL3 conditions at the University of Washington BSL3/ABSL3 High Containment Facility.

### Studies in mice

hCD46-transgenic mice are C57Bl/6-based transgenic mice that contain the human CD46 genomic locus and provide CD46 expression at a level and in a pattern similar to humans were described earlier.^32^ K18-hACE2-transgenic mice [B6.Cg-Tg(K18-ACE2)2Prlmn/J] from the Jackson Laboratory can be infected with SARS-CoV2 virus, resulting in severe illness characterized by weight loss, rapid breathing, hunched posture, and inactivity. In addition, viral infection results in lesions in the lungs determined by postinfection pathology, as well as chemokine/cytokine storm traits observed in human patients and viral replication in the gut and heart. The human keratin 18 promoter directs expression to epithelia, including airway epithelia where infections typically begin.

#### HSC mobilization and *in vivo* transduction

This procedure was described previously.^3^ Briefly, HSCs were mobilized in mice by s.c. injections of human recombinant G-CSF (5 μg/mouse/day, 4 days; Amgen, Thousand Oaks, CA) followed by an s.c. injection of AMD3100 (5 mg/kg; Sigma-Aldrich) on day 5. In addition, animals received dexamethasone (10 mg/kg) i.p. 16 and 2 h before virus injection. Thirty and 60 min after AMD3100, animals were intravenously injected with HDAd-sACE2-Ig and HDAd-SB (1:1 ratio) through the retro-orbital plexus with a dose of 4 × 10^10^ vp for each virus per injection.

Following treatment, combined immunosuppression was administered. At week 4, mice were subjected to five cycles of *in vivo* selection with O^6^BG (30 mg/kg, i.p.) and escalated BCNU doses (5, 7.5, 10, 10, and 10 mg/kg) with a 2-week interval between doses. Immunosuppression was resumed 2 weeks after the last O^6^-BG/BCNU dose.

#### Immunosuppression

Intraperitoneal injection of a mycophenolate mofetil (20 mg/kg/day), rapamycin (0.2 mg/kg/day), and methylprednisolone (20 mg/kg/day) was performed every other day.

#### Generation of sACE2-Ig-expressing K18-hACE2tg mice

Lineage-negative cells from *in vivo* HDAd-transduced CD46tg mice were transplanted into female K18-hACE2tg. On the day of transplantation, recipient mice were irradiated with 1,000 Rad. Bone marrow cells from *in vivo* transduced CD46tg mice were isolated aseptically and lineage-depleted cells were isolated using magnetic-activated cell sorting. Six hours after irradiation, cells were injected intravenously at 1 × 10^6^ cells per mouse.

#### SARS-CoV2 inoculation

K18-hACE2tg transplanted with bone marrow lin^−^ cells from mock (control) or HDAd-sACE2-Ig (test)-transduced mice were transferred to the University of Washington BSL3 facility.

Mice were challenged intranasally with 10^3^ PFU (plaque-forming units) SARS-COV-2 WA1 (BEI NR-53872) and body weight was monitored daily. At indicated time points, a subset of mice was sacrificed and tissue samples were collected for analysis. Otherwise, mice were sacrificed at 20% loss of starting weight and survival curves generated. Blood was collected, centrifuged at 2,000 *g* for 10 min, and serum was aliquoted. Trizol was added to blood cells. Right lungs were collected, and lobes separated into PBS for determination of viral titers and Trizol for analysis of viral RNA copies. Lungs were homogenized in lysing tubes with a Precellys 24 homogenizer.

Homogenized samples were centrifuged at 2,000 *g* for 10 min and supernatant collected. All processed samples were stored at −80°C until analysis by plaque assay or qRT-PCR. Left lung was collected and fixed in 10% neutral-buffered formalin for 3 days before transfer to 70% EtOH and processing for paraffin embedding and sectioning (University of Washington Histology Core).

### Studies in NHPs

A female rhesus macaque (*Macaca mulatta*) from the Oregon National Primate Research Center was used for the studies. After implantation of an intravenous catheter, the animal was housed individually.

### Mobilization

Filgrastim/G-CSF/Neupogen (Amgen): 50 mcg/kg, subcutaneously (SQ), start SID (once daily) on day 5 afternoon, daily SID in afternoon through day 0; and AMD3100/Plerixafor (Calbiochem) (5 mg/kg, SQ SID midnight on days 1 and 0; doses are given at midnight before HDAd5/35^++^ dosing at 8am).

### Cytokine prophylaxis

Dexamethasone (4 mg/kg, first dose IV SID 2pm on day 2, two doses each day 1 and 0; Fresenius Kabi USA), Anakinra/Kineret (Swedish Orphan Biobivitrium): 50 mg/dose-flat dose, two doses each SQ day 1 and 0 (1 h before and 6 h after HDAd5/35^++^ injection), one dose each day 1 and 2; and Tocilizumab/Actemra (Genentech, Inc.) (8 mg/kg, two doses IV each day 1 and 0; 1 h before and 6 h after HDAd injection), diluted infusions IV (50 mL each).

### HDAd5/35^++^ vector infusion

For infusion, HDAd preps are thawed, diluted with PBS (room temperature), and infused within 30–60 min after preparation. A low dose (5 × 10^10^ vp/kg) in 5 mL of PBS is given over 10 min followed by the therapeutic dose (usually 1.6 × 10^12^ vp/kg) in 20 mL of PBS infused over 20 min.

### Immunosuppression

Tacrolimus/PRPGRAF (Astellas Pharma): 0.02 mg/kg to start, then adjust to get blood level ∼15 ng/mL, start SQ BID (bis in die-twice a day) on day 5 morning, continue through study. Mycophenolate Mofetil Hydrochloride/MMF/CellCept (Genentech): 40 mg/kg, start day 1, 20 mg/kg PO BID; Orencia/Abatacept (Brystol-Myers Squibb): 60 mg/dose-flat dose, IV doses on days 2, 0, 2, 7, and 14.

### *In vivo* selection

O^6^-Benzylguanine (Sigma) and Carmustine/BCNU (Sigma) were made fresh for each injection. First, O^6^BG (120 mg/m^2^) is infused IV over 15 to 20 min (flow rate ∼600 mL/h). BCNU is given ∼30–45 min after the end of O^6^BG infusion. The BCNU doses were 10, 20, and 30 mg/m^2^. O^6^BG infusion (120 mg/m^2^) is repeated 7–8 h after the end of the first infusion. Neutrophil counts decreased after each round of O^6^BG/BCNU treatment. The subsequent dose of O^6^BG/BCNU was therefore given only after neutrophil counts recovered (2–4 weeks).

### Flow cytometry gating of GFP-positive CD34^+^/CD45RA^−^/CD90^+^ cells

The following antibodies were used: CD34APC (563 Clone): Cat# 561209 from BD; CD90 PE (5e10 Clone): Cat # 328110 from BioLegend; CD45RA APCH7 (5H9 Clone): Cat# 561212 from BDCD45NHP; and BV421 (D0581283 Clone): Cat# 561291 from BD.

### Cytometric bead array

The NHP Th1/Th2 cytokine CBA kit (BD Biosciences 557800) was used to measure serum levels for IL-2, IL-4, IL-5, IL-6, tumor necrosis factor (TNF), and interferon gamma (IFNγ).

### sACE2-Ig antibody ELISA (rhesus serum)

Plates were coated with recombinant proteins sACE-Ig (0.3 μg/well in 0.1 M Na-bicarbonate buffer pH9.6, o/n at 4°C). Serum serial dilutions (starting at 1:50, 3 × subsequent dilutions) were added for 1 h. After washing, HRP-conjugated secondary antibodies against the HA-tag were plated at 1:10,000 for 1 h. After wash, Thermo 1-Step Ultra TMB Solution (ThermoFisher Scientific) was added, and the color was allowed to develop for 7 min before stopping with 2 N sulfuric acid. Absorbance readings at 450 nm were taken with a plate reader. Antibody reactivity curves were plotted with GraphPad Prism with a four-parameter curve, and EC_50_ values were accordingly calculated.

### Statistical analyses

For comparisons of multiple groups, one-way and two-way analysis of variance (ANOVA) with Bonferroni post-testing for multiple comparisons were employed. Statistical analysis was performed using GraphPad Prism version 6.01 (GraphPad Software, Inc., La Jolla, CA).

## Results

### HDAd-ACE2-Ig vector

**Figure 1.**
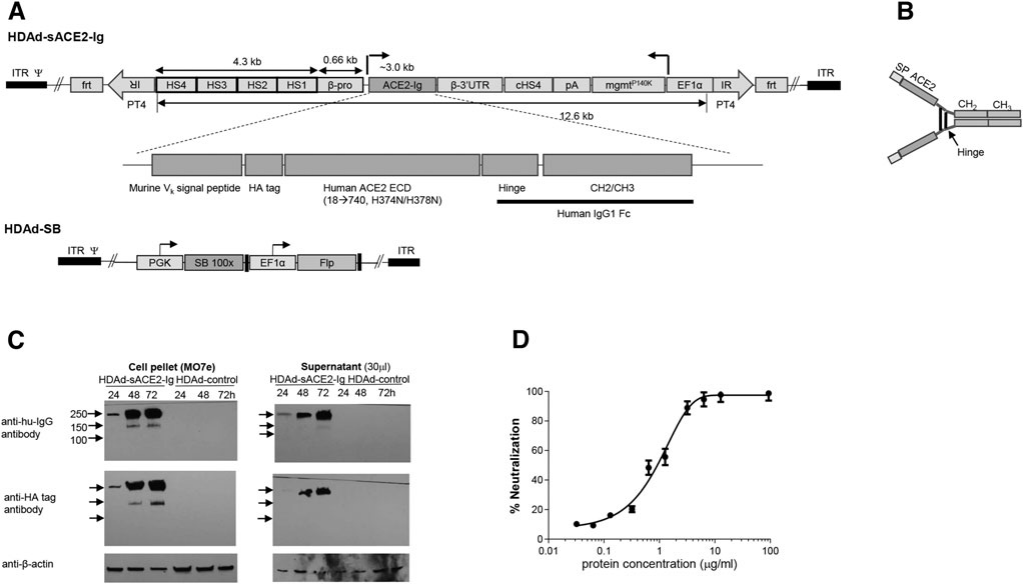
HDAd5/35^++^ vector expressing sACE2-Ig. **(A)** Vector structure. In HDAd-sACE2-Ig, the 12.6 kb transposon is flanked by optimized inverted transposon repeats (T4 IR) and FRT sites. The sACE2-Ig expression cassette contains a 4.3 kb version of the human β-globin LCR consisting of four DNAse HS regions and β-globin promoter. The cDNA encoding secreted, extracellular domain of human ACE2 fused to human constant IgG(γ1) domains was synthesized and inserted after LCR/β-promoter, two active-site histidine residues were mutated (H374N and H378N) to reduce the catalytic activity of ACE2. HA tag was inserted in the N-terminal for detection. The human β-globin 3′UTR (for mRNA stabilization in erythrocytes) was used. The vector also contains an expression cassette for MGMT^P140K^ allowing for *in vivo* selection of transduced HSPCs and HSPC progeny. The bidirectional SV40 poly-adenylation signal is used to terminate transcription. To avoid interference between the LCR/β-promoter and EF1α promoter, a 1.2 kb chicken HS4 chromatin insulator was inserted between the cassettes. **(B)** Antibody-like structure of sACE2-Ig. **(C)** *In vitro* expression. Erythroid MO7e cells were transiently transduced with HDAd-sACE2-Ig vector at an MOI of 1,500 vp/cell. Cell pellet and culture medium (for secreted sACE2-Ig) were collected at 24, 48, and 72 h postinfection. The figure shows Western blots with either HA-tag antibody (N terminal) or human IgG(Fc) antibody (C terminal). β-Actin was used as loading control. **(D)** Recombinant ACE2-Ig protein neutralizes lentiviruses pseudotyped with SARS-CoV2 spike protein. SARS-CoV2-S protein-pseudotyped lentivirus expressing firefly luciferase was incubated with increasing concentrations of purified sACE2-Ig protein for 1 h before infection of HEK293T cells expressing ACE2 (293/ACE2). Luciferase activities were measured 60 h after infection and the percent of neutralization was calculated. HA, influenza hemagglutinin tag; HS, hypersensitivity; LCR, locus control region; UTR, untranslated region.

We aimed to continuously express the SARS CoV2 decoy receptor sACE2-Ig from erythrocytes, the most abundant cells in the body. Theoretically, this could block viremia and spreading of infection in the lung. To allow for high-level sACE2-Ig expression in erythroid cells, we employed a 5 kb version of the β-globin LCR to drive transgene expression and the β-globin 3′UTR (untranslated region) for mRNA stabilization (Fig. 1A).

The human sACE2 extracellular domain^33^ was fused on its N-terminus with an Ig kappa chain signal peptide to mediate secretion and, on its C-terminus with a human IgG1 Fc region to improve pharmacokinetics of the protein. The signal peptide will be cut off in the endoplasmic reticulum. The HA tag will still be present in the secreted protein. The protein chain dimerizes through the hinge region, thereby forming an antibody-like structure (Fig. 1B). The vector also contained an *mgmt^P140K^* expression cassette that can mediate the expansion of transduced HSPCs by O^6^BG/BCNU.^8^ The HDAd-sACE2-Ig vector is targeted to CD46, a membrane protein that is expressed at high levels on primitive HSCs.^3^

The sACE2-Ig/mgmt cassette-containing transposon can be integrated randomly into the genome of transduced cells by a hyperactive *Sleeping Beauty* transposase (SB100x) expressed from a second HDAd5/35^++^ vector, HDAd-SB (Fig. 1A). In HDAd-sACE2-Ig, we used T4 inverted repeats (IRs) that had been engineered to increase the SB100x transposition and integration rate.^34^ In this study, we confirmed that the T4 IRs are superior over previous IR generations (T0^7^ and T2^35^) in mediating integration of a transposon (Supplementary Figs. S1 and S2). Notably, we used a catalytically inactive form of sACE2-Ig, in which residues involved in binding angiotensin I and angiotensin II were mutated to avoid perturbing the renin–angiotensin–aldosterone system.^33^

### *In vitro* tests

To test the functionality of the HDAd-sACE2-Ig vector, we infected an erythroleukemia cell line (MO7e) and evaluated sACE2-Ig in the cell pellet and culture supernatant by Western blot with antibodies specific to the Fc domain and HA-tag. Both probes detected an ∼250 kDa protein in both samples, indicating efficient expression and secretion of sACE2-Ig (Fig. 1C). As a positive control for functional assays, we generated purified recombinant sACE2-Ig using the same transgene that is present in our HDAd vectors. The protein was expressed in 293 cells and purified from the supernatant using protein A resin.

Recombinant sACE2-Ig was subjected to a functional assay that measured the ability to neutralize a SARS-CoV2 surrogate. This assay included a luciferase-expressing retrovirus that contained the SARS-CoV2 spike protein instead of the native ENV protein and 293 cells that expressed the ACE2 receptor on their surface.^36^

The S protein-pseudotyped lentivirus did not infect control 293 cells without the ACE2 receptor (data not shown). The concentration of recombinant sACE2-Ig that inhibited 50% lentivirus infection/luciferase expression (IC_50_) was about 800 ng/mL (Fig. 1D). This level of activity is comparable to that reported by Liu *et al.*,^37^ which implies that the sACE2-Ig gene used in our HDAd vectors is functionally active. We used the recombinant sACE2-Ig protein for a standard curve in an ELISA to measure sACE2-Ig concentrations in *in vivo* transduced mice and NHP.

### *In vivo* HSC transduction in CD46-transgenic mice

**Figure 2.**
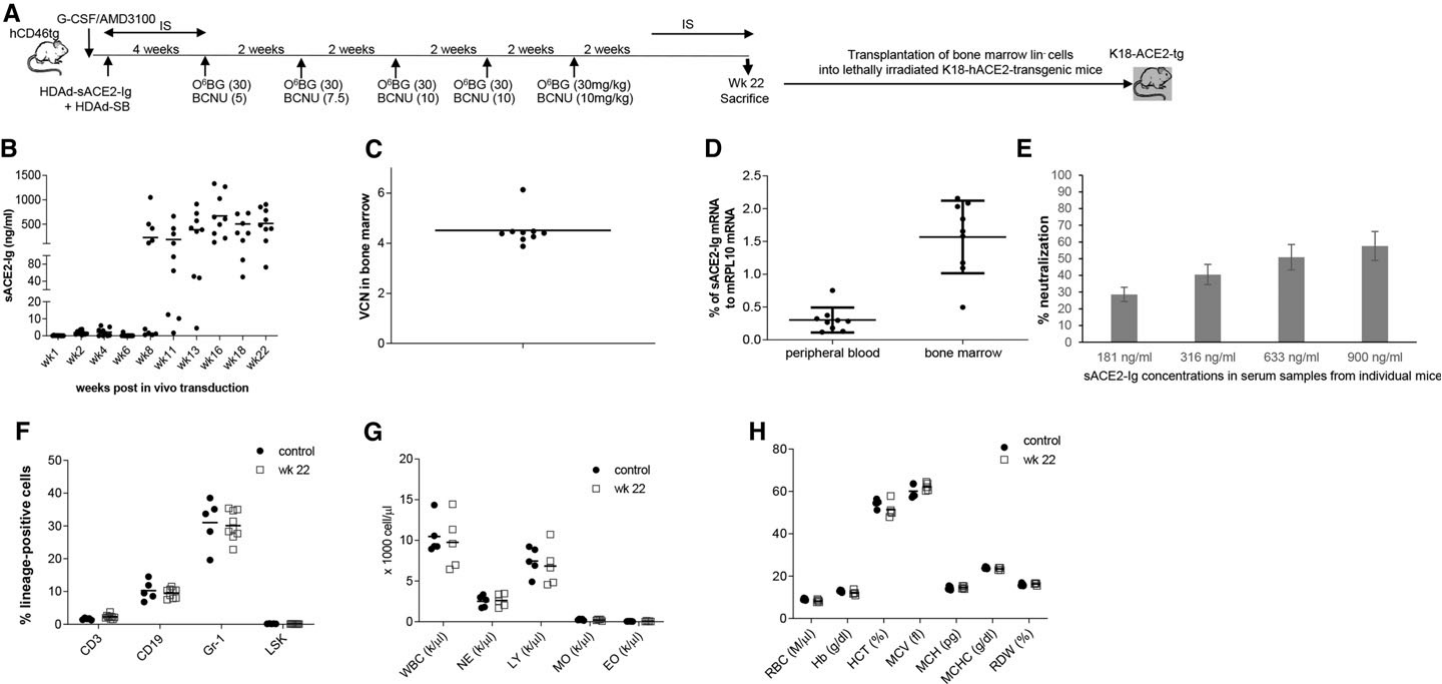
*In vivo* HSPC transduction in hCD46tg mice. **(A)** *In vivo* transduction of mobilized hCD46tg mice. HSCs were mobilized by s.c. injections of human recombinant G-CSF for 4 days followed by one s.c. injection of AMD3100. Thirty and 60 min after AMD3100 injection, animals were injected IV with a 1:1 mixture of HDAd-ACE2-Ig plus HDAd-SB (two injections, each 4 × 10^10^ viral particles). Mice were treated with IS drugs for the next 4 weeks to avoid immune responses against the human ACE2-Ig and MGMT^P140K^. O^6^BG/BCNU treatment was started at week 4 and repeated every 2 weeks five times. The BCNU concentration was slowly increased, from 5 to 7.5 to 10 mg/kg. Immunosuppression was resumed 2 weeks after the last O^6^BG/BCNU injection. Mice were followed until week 22, when animals were sacrificed for analysis. BM lin^−^ cells were transplanted into lethally irradiated K18-hACE2-transgenic mice. **(B)** sACE2-Ig protein levels in serum measured by ELISA. The ELISA consisted of S1 protein for capture and anti-HA tag antibodies for detection. Each *symbol* is an individual animal. **(C)** VCN per cell in BM mononuclear cells at week 22 after *in vivo* transduction. **(D)** sACE2-Ig mRNA levels in peripheral blood RBCs and BM of week 22 measured by qRT-PCR relative to mouse mRPL10 mRNA levels. **(E)** Neutralization assay with serum sACE2-Ig. Week 22 serum samples from mice were incubated with the S protein-pseudotyped lentivector for 1 h before infection of 293/ACE2 cells. Luciferase activity was measured 60 h after infection and the percentage of neutralization was calculated. Shown are data for four mice with the different serum sACE2-Ig concentration shown below the X-axis. **(F)** Cellular BM composition in control and treated mice euthanized at week 22. Shown is the percentage of lineage marker-positive cells (CD3^+^, CD19^+^, and Gr-1^+^), as well as LSK cells. **(G)** Blood cell counts in control and hCD46-transgenic mice at week 22 after *in vivo* HSC transduction. **(H)** Hematological parameters. The differences between the two groups were not significant. BCNU, Carmustine; BM, bone marrow; ELISA, enzyme-linked immunosorbent assay; G-CSF, granulocyte colony-stimulating factor; HSC, hematopoietic stem cell; IS, immunosuppressive; qRT-PCR, real-time quantitative reverse transcriptase PCR; RBC, red blood cell; VCN, vector copy number.

*In vivo* HSC transduction with HDAd5/35^++^ vectors requires CD46 as a receptor. The biodistribution and function of CD46 in mice and humans are different.^38^ In humans, CD46 is present on all nucleated cells, while the expression of the mouse CD46 ortholog is restricted to the testis. For our *in vivo* HSC transduction studies, we therefore used human CD46-transgenic mice. These mice carry 400 kb of the human CD46 locus^39^ and express the protein in a pattern similar to humans.^32^ For *in vivo* transduction in CD46-transgenic mice, HSCs were mobilized with G-CSF/AMD3100 and intravenously injected with HDAd-sACE2-Ig plus HDAd-SB (Fig. 2A).

Four weeks later, mice were subjected to O^6^BG/BCNU *in vivo* selection to expand sACE2-Ig-expressing erythroid progenitor cells. Serum sACE2-Ig levels reached 500–1,300 ng/mL after the fifth cycle of selection (Fig. 2B). At week 22, *in vivo* transduced mice were euthanized and bone marrow lineage-negative (lin^−^) cells (which are enriched for HSCs) were transplanted into lethally irradiated K18-hACE2 mice to create an sACE2-Ig-expressing mouse model that is susceptible to SARS-CoV2 infection (see section “CoV2 challenge studies in sACE2-Ig expressing K18-hACE2-transgenic mice”).

Furthermore, more detailed analyses were performed with blood and bone marrow samples collected at week 22 from *in vivo* transduced (primary) CD46tg mice.

The average VCN per cell in bone marrow mononuclear cells (MNCs) was 4.5. indicating efficient *in vivo* transduction (Fig. 2C). sACE2-Ig mRNA levels, measured by qRT-PCR relative to a mouse mRPL10 mRNA, were ∼7-fold higher in total bone marrow cells compared to total blood cells. Considering that the vast majority of cells in blood and bone marrow are erythroid cells, this implies that sACE2 is predominantly produced from erythroid cells in the bone marrow (Fig. 2D), which is in agreement with our previous data showing that the β-globin LCR is not active in nonerythroid cells.^12^ This is desirable because nucleated erythroid progenitors have a more potent biosynthetic and secretory activity than enucleated peripheral red blood cells.

The ability to neutralize S protein-pseudotyped lentivector was measured in week 22 serum of mice with different serum sACE2-Ig levels (Fig. 2E). The results agree with our study that used recombinant sACE2-Ig; the IC_50_ was about 800 ng/mL. At a concentration of 181 ng of sACE2-Ig per milliliter serum, about 30% of infection was blocked. This shows that sACE2-Ig produced from erythroid cells is functionally active. High-level continuous sACE2 expression in *in vivo* transduced CD46-transgenic mice had no detrimental effects on body weight and hematological parameters, which were comparable to nontransduced CD46-transgenic mice (Fig. 2F–H).

### CoV2 challenge studies in sACE2-Ig-expressing K18-hACE2-transgenic mice

**Figure 3.**
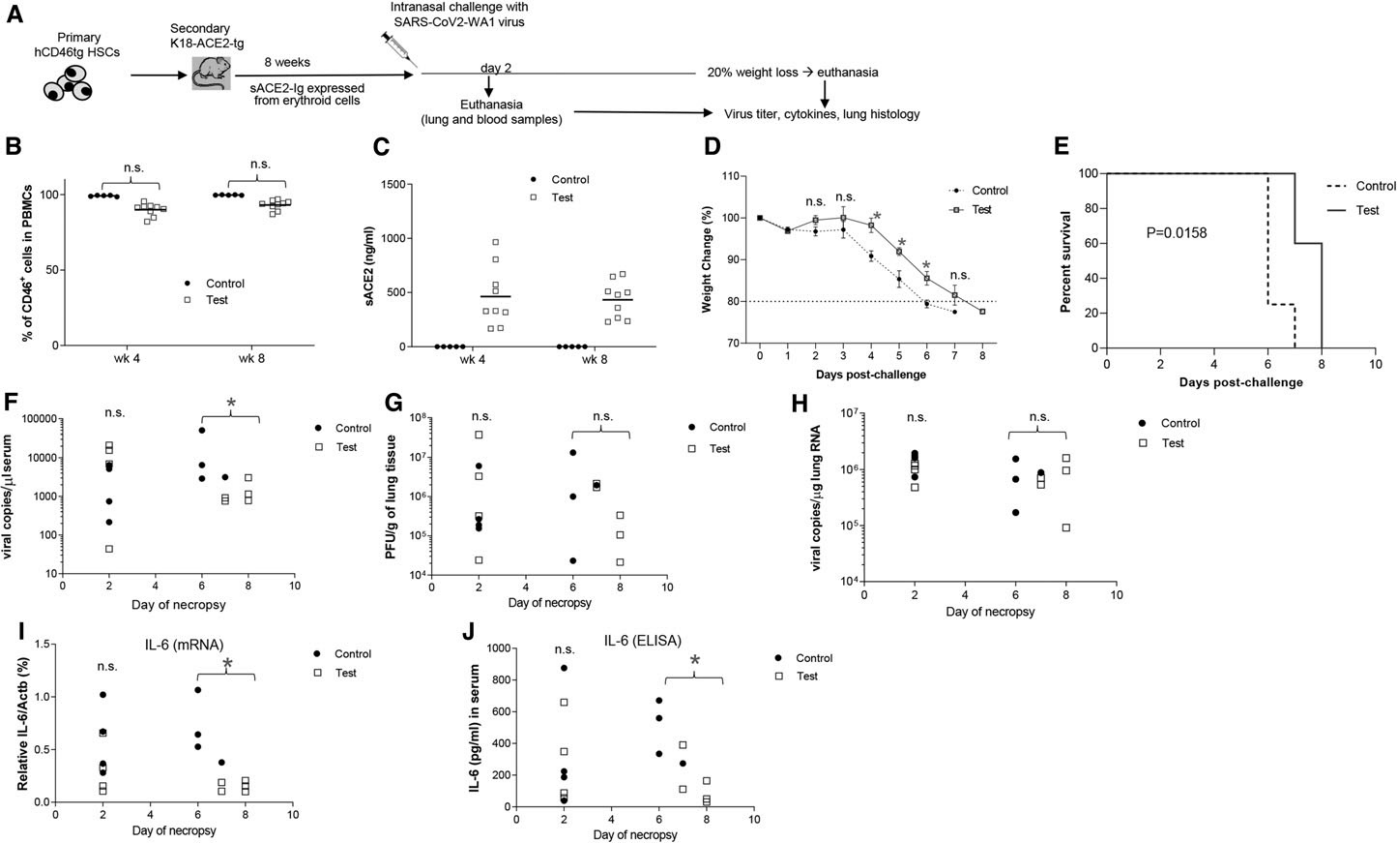
SARS-CoV2 challenge studies in K18-hACE2-expressing sACE2-Ig. **(A)** Experimental scheme. BM Lin^−^ cells from primary *in vivo* transduced hCD46tg mice were transplanted into lethally irradiated K18-hACE2-transgenic mice. Lin^−^ cells from HDAd-sACE2-Ig vector *in vivo* transduced mice were used for the test group (“Test”). Lin^−^ cells from mice *in vivo* transduced with a control HDAd5/35^++^ vector were used for the control groups (“Control”). The transplanted K18-hACE2 mice were then followed for 8 weeks before they were challenged by intranasal inoculation of lethal dose of SARS-CoV2 virus. Four mice from each group were sacrificed on day 2 after challenging. The other mice were sacrificed when their body weight loss reached 20%. Lung and blood samples were collected for further analysis. **(B)** Engraftment rate of both control and test K18-hACE2 mice at week 4 and 8 after transplantation. **(C)** Serum sACE2-Ig levels in both control and test group measured by ELISA. **(D)** Body weight loss in mice after SARS-CoV2 inoculation performed on day 0. Shown is the percentage of weight loss relative to body weights taken immediately before challenge. **(E)** Kaplan-Meier survival curves of mice. The difference between the groups is significant. **(F–H)** Viral loads. **(F)** CoV2 mRNA copies in serum measured by qRT-PCR. The difference between the test and control group was significant (*p < 0.05 for “Control” vs. “Test” mice that had reached the endpoint between days 6 and 8). **(G)** Viral load in lung samples measured by SARS-CoV plaque assay. Shown are PFU per gram lung tissue. **(H)** Number of viral RNA copies per μg total lung tissue RNA measured by qRT-PCR. **(I, J)** IL-6 levels. **(I)** IL-6 mRNA levels relative to β-actin mRNA level in lung tissue samples measured by qRT-PCR (*p < 0.05 for “Control” vs. “Test” mice that had reached the endpoint between days 6 and 8). **(J)** IL-6 serum levels measured by ELISA (*p < 0.05 for “Control” vs. “Test” mice that had reached the endpoint between days 6 and 8). IL, interleukin; n.s., nonsignificant; PFU, plaque-forming units.

To create a mouse model that is susceptible to SARS-CoV2 (WA-1) infection, week 22 bone marrow lineage-negative cells from *in vivo* transduced mice were transplanted into lethally irradiated K18-ACE2-transgenic mice (Fig. 3A). Notably, all blood cells in these mice are derived from HSCs of the *in vivo* transduced CD46tg mice, while the lung epithelium expresses the human ACE2 receptor. The control group was K18-ACE2 mice transplanted with lin^−^ cells harvested from mice at week 22 after mobilization and control HDAd injection. Engraftment of transplanted HSCs was not significantly different for both groups based on the percentage of peripheral blood human-CD46^+^ cells (Fig. 3B).

The serum sACE2-Ig levels at weeks 4 and 8 were stable in the range of 230–680 ng/mL (Fig. 3C). At week 8 after transplantation, animals were intranasally inoculated with a lethal dose of the CoV2/WA-1 strain. In comparison to the inoculated control K18-ACE2 mice group, a significant prevention of weight loss was found in the test group on days 4, 5, and 6 (Fig. 3D). With an endpoint defined as 20% weight loss, sACE2-Ig-expressing mice survived significantly longer after the CoV2 challenge (Fig. 3E). Viral load was analyzed in blood and lung tissue in mice euthanized on day 2 after virus inoculation and on the day when the mice reached the endpoint/day of euthanasia (*i.e.,* days 6, 7, or 8) (Fig. 3F–H).

**Figure 4.**
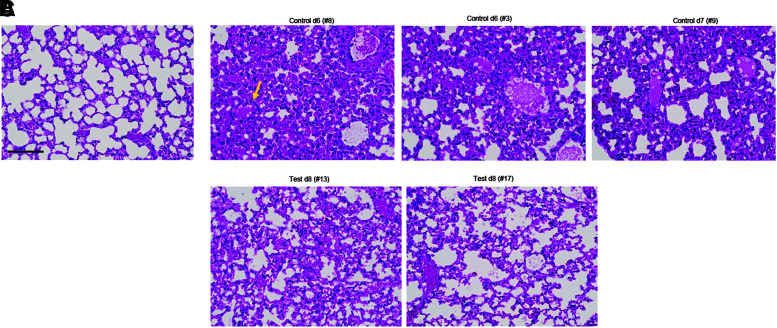
Analysis of secondary K18-hACE2 mice expressing sACE2-Ig from erythroid cells: lung histology. **(A)** Lung section of a healthy mouse with clear alveoli lined by single column epithelial cells. **(B)** Sections from three mice from the control group taken at the time of euthanasia (days 6 and 7) display diffuse alveolar damage, massive infiltration of leukocytes, and the presence of scattered large protein globules (see *yellow arrow*). **(C)** Signs of lung damage and inflammation are less pronounced in sections from mice of test group taken at the end of the study (day 8). The scale bar is 20 μm. Representative sections are shown.

Serum CoV2 mRNA levels measured by qRT-PCR were significantly lower in the test group, suggesting that sACE2-Ig reduced viremia (Fig. 3F). Viral loads in lung samples were measured by plaque assay for infectious virus (Fig. 3G) and qRT-PCR for viral mRNA (Fig. 3H). While there was a tendency toward lower viral titers in the test group, the difference was not significant. In lung samples, there were significantly lower levels of IL-6 (a key marker for the CoV2-induced lung inflammation^40^) in the test animals, both at the mRNA and protein levels (Fig. 3I, J). Along this line, lung sections of the test group showed less histopathological signs of alveolar inflammation and damage that are characteristic for an acute COVID-19 infection in humans (Fig. 4).

In summary, sACE2-Ig expressed from erythroid cells diminishes SARS-CoV2 sequelae in mice. Treated mice lost less weight, had less viremia, and displayed reduced cytokine production and lung pathology. The therapeutic effects are not apparent in animals euthanized on day 2, but becomes pronounced at later time points, indicating that the challenge load was too high to be controlled at the level of primary infection, and that sACE2-Ig rather acts at viremic spread of *de novo* produced virus.

### Safety of continuous sACE2-Ig expression after in vivo HSC transduction in a rhesus macaque

In the mouse studies, we did not observe clinical or hematological side effects of continuous sACE2-Ig expression from erythroid cells. The mouse model, however, has limitations because human sACE2-Ig might not be able to trigger downstream signaling as it would in humans. A better model is nonhuman primates. The amino acid homology between the human and rhesus ectodomain of ACE2 is 95%, while the homology is only 83% for mouse versus human. Rhesus macaques were used in SARS-CoV2 therapy studies with neutralizing monoclonal antibodies and sACE2-Ig protein.^41^

Recently, we have applied our *in vivo* HSC transduction/selection approach in rhesus macaques using γ-globin expressing HDAd5/35^++^ vectors.^14^ To expand these studies with a secreted protein and to collect more safety data for a potential clinical translation of our approach, we performed *in vivo* HSC transduction with our HDAd-sACE2-Ig vector in a rhesus macaque, which we then followed for 17 weeks. Because our transgene products were human and the test animal is fully immunocompetent, we applied immunosuppression consisting of daily tacrolimus and MMF.

**Figure 5.**
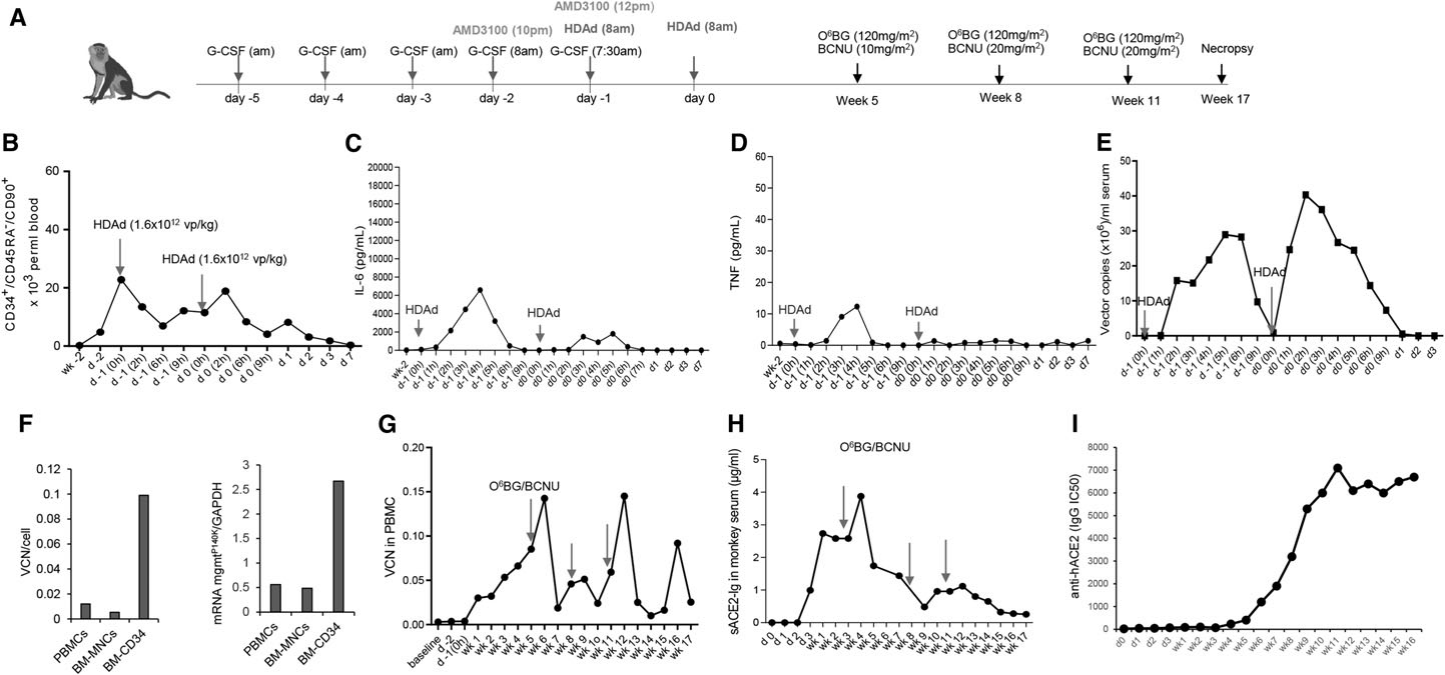
*In vivo* HSC transduction in a rhesus macaque with HDAd-sACE2-Ig. **(A)** Treatment regimen. A female rhesus macaque was mobilized by s.c. injections of human recombinant G-CSF and AMD3100. At the peak number of HSCs in the peripheral blood after AMD3100 injection, animal was injected IV with a 1:1 mixture of HDAd-ACE2-Ig plus HDAd-SB (two injections, each 1.6 × 10^12^ vp/kg). Immediately before each high-dose (1.6 × 10^12^ vp/kg) injection, the animal was infused with a “low” vector dose (5 × 10^10^ vp/kg) in an attempt to saturate cells that nonspecifically sequester HDAd particles. O^6^BG/BCNU treatment was applied at weeks 5, 8, and 11. **(B)** Numbers of primitive (CD34^+^/CD45RA^−^/CD90^+^) HSCs in peripheral blood. HDAd vector injections are marked by *red arrow*. The time of HDAd injection is indicated as “0 h” correspondingly on days 1 and 0. After this time point, blood samples were analyzed at 2, 6, and 9 h on days 1 and 0 and then, on days 1, 2, 3, and 7. **(C, D)** Serum levels of proinflammatory cytokines after HDAd injection. The animal received pretreatment with dexamethasone, tocilizumab, and anakinra. **(C)** IL-6 levels measured by cytometric bead array. **(D)** TNFα levels measured by CBA. IL-2, IL-4, IL-5, and IFNγ were not detectable. **(E)** HDAd vector genome copies in serum samples were measured by qPCR. **(F)** HDAd genomes in PBMCs. Shown is the VCN per cell. Treatment with O^6^BG/BCNU is indicated by *red arrows*. The BCNU doses were 10, 20, and 20 mg/m^2^. **(G)** Long-term analysis of HDAd genomes in PBMCs. Treatment with O^6^BG/BCNU is indicated by *red arrows*. **(H)** sACE2-Ig serum levels measured by ELISA. **(I)** Serum titers of IgG anti-sACE2-Ig antibodies (IC_50_ titers). IFNγ, interferon gamma; PBMCs, peripheral blood mononuclear cells; TNF, tumor necrosis factor.

HSCs were mobilized from the bone marrow by subcutaneous administration of G-CSF/plerixafor, and while they were in the periphery, they were transduced by intravenously injected HDAd5/35^++^-sACE2-Ig+HDAd5/35^++^-SB at total dose of 3.2 × 10^12^ vp/kg given on 2 days (days 1 and 0) (Fig. 5A). Mobilization efficacy was measured by flow cytometry for primitive HSCs (CD34^+^/CD45RA^−^/CD90^+^ cells) present in peripheral blood and was in the range of ∼20 × 10^3^ cells/mL peripheral blood at the time of vector injection (Fig. 5B).

Intravenous HDAd5/35^++^ injection at this dose was well tolerated after appropriate cytokine prophylaxis (dexamethasone, IL-6R monoclonal antibody tocilizumab, and IL-1 blocker anakinra). Serum IL-6 and TNF cytokine levels were in the noncritical range (Fig. 5C, D). (Considered critical are IL-6 levels >35 ng/mL.^42^) The number of vector genomes per milliliter serum was measured by qPCR using *mgmt^P140K^*-specific primers (Fig. 5E).

At 2 h after each intravenous HDAd5/35^++^ injection, vector genomes per milliliter serum were in the range of 30–40 × 10^6^. By 10 h, the vast majority of vector genomes had been cleared from the circulation. To demonstrate *in vivo* HSC transduction, we measured VCN on day 7 in PBMCs, total bone marrow MNCs, and bone marrow CD34^+^ cells (Fig. 5F). The data indicate preferential *in vivo* transduction of CD34^+^ HSCs, which is most likely due to higher density of the HDAd5/35^++^ receptor CD46 on HSCs.^3^ Long-term analysis of VCN in PBMCs indicated that O^6^BG/BCNU selection expanded transduced cells; however, overall, the trend was toward a decline of vector-transduced PBMCs (Fig. 5G).

The key readout from this study, serum sACE2-Ig levels (measured by ELISA) showed a similar complex pattern. After a continuous rise over 5 weeks, sACE2-Ig levels dropped and could not be salvaged by the second and third round of *in vivo* selection (Fig. 5H). Concurrent with the decline of serum sACE2-Ig concentration measured by ELISA, the IgG titer of serum antibodies against human ACE2 increased in this animal (Fig. 5I). This indicates that immunosuppression was insufficient and anti-sACE2-Ig antibodies that developed interfered with the detection of serum sACE2-Ig and/or that T cell responses destroyed sACE2-Ig-producing erythroid cells.

**Figure 6.**
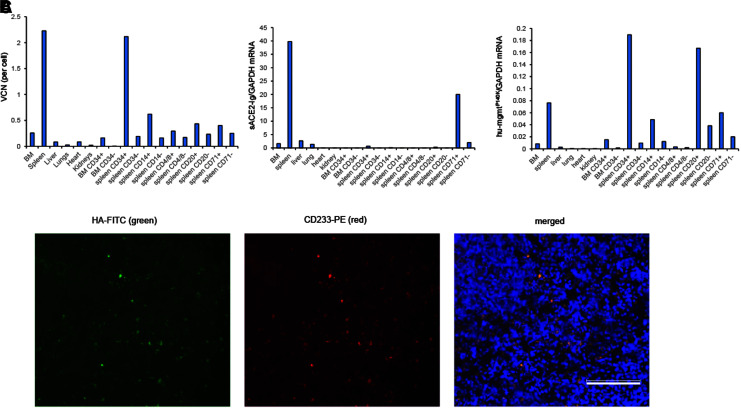
Vector biodistribution and safety in the rhesus macaque. **(A)** Week 17 vector biodistribution in major organs and in CD34, CD14, CD20, and CD71 subsets of BM and spleen. Shown is the VCN per cell. **(B)** Relative sACE-Ig mRNA (*left panel*) and human mgmt^P140K^ mRNA (*right panel*) levels in tissues, BM, and spleen subsets. **(C)** Expression of sACE2-Ig in erythroid cells of the spleen. Anti-HA tag antibodies (*green*) were used to detect sACE2-Ig. Anti-CD233 antibodies (*red*) detected erythroid cells.

Biodistribution studies performed at week 17 showed the highest VCN (∼2 copies per cell) in the spleen and within the analyzed spleen cell subsets, in splenic CD34^+^ cells (Fig. 6A). sACE2-Ig mRNA was also highest in the spleen (Fig. 6B, left panel).

Because sACE2-Ig is expressed from a β-globin LCR version, the highest sACE2-Ig mRNA levels were found in erythroid cells (CD71^+^ cells). On the other hand, mgmt^P140K^ mRNA expressed from the ubiquitously active EF1a promoter is also produced in the other subsets, predominantly in CD20^+^ cells (Fig. 6B, right panel). The presence of sACE2-Ig (HA-tag) in erythroid (anti-CD233/Band 3^+^) cells was also demonstrated by immunofluorescence analysis of spleen sections (Fig. 6C). This indicates that mobilized *in vivo* HDAd5/35^++^-transduced HSCs preferentially localize to and survive in the spleen and that sACE2-Ig is expressed from erythroid cells in the spleen.

A major goal of this NHP study was to assess potential side effects of long-term sACE2-Ig expression. Because of immune responses, sACE2-Ig was detectable in serum for about 5 weeks. During that period (and also for the length of the 17-week study), none of our hematology, pathology, and histopathology evaluations indicated remarkable side effects (Supplementary Fig. S3). The 10% weight loss that was observed is common for animals on intravenous tether.

Importantly, because our HDAd5/35^++^ vector was designed not to transduce hepatocytes, liver transaminases remained within the normal range. As seen in mice, sACE2-Ig production in erythroid cells did not affect red blood cell parameters. As expected, G-CSF/AMD3100 mobilization triggered transient leukocytopenia that normalized by day 7. Also, related to mobilization and HDAd5/35^++^ injection, a mild, transient thrombocytopenia was observed during the first 4 days. In the bone marrow, G-CSF/AMD3100 mobilization increased the fraction of CD14^+^ cells and decreased the percentage of CD3^+^ cells, both of which returned to normal levels by week 1.

Overall, our NHP study suggests that *in vivo* HSC transduction is safe and that sACE2-Ig expression in erythroid cells does not affect erythropoiesis and the function of erythrocytes.

## Discussion

SARS-CoV2 variants may continue to adapt in the human population for years or decades. The S protein is rapidly diversifying, potentially necessitating the adjustment of vaccines to newly emerging variants. HSC gene therapy with secreted decoy receptors of viral infections is a recognized strategy for HIV prophylaxis and therapy.^43–45^ In this study, we extended this idea to COVID-19 by employing a new *in vivo* HSC transduction approach.

Potential advantages include the following: (i) the continuous production of the common SARS-CoV receptor decoy will be broadly protective against virus escape mutants because it is highly unlikely that the virus will switch its primary receptor. (ii) Stable, high serum levels of recombinant sACE-2-Ig might intervene with viremia, thereby preventing the large spectrum of acute and chronic symptoms that are not directly related to lung infection. (iii) The approach can be used in individuals that cannot be vaccinated (*e.g.,* due to anaphylactic reactions) or that are immunosuppressed (*e.g.,* cancer patients under chemotherapy or HIV/AIDS patients).

In these cases*, in vivo* sACE2-Ig expression could be combined with HSC gene therapy for cancer^13^ and AIDS^46^ by incorporating corresponding effector gene expression cassettes into the HDAd vector (which has 35 kb insert capacity).

Clearly, for a widespread application, such an approach must be technically simple. We are developing an *in vivo* gene therapy approach that requires only intravenous injections and can be completed in 1 day. It would lead to continuous expression of a SARS-CoV2 decoy receptor from erythroid cells, the most abundant cell type in body to achieve high systemic and local protein levels.

Compared to a recently published gene therapy approach that involves the intranasal inoculation of an AAV vector expressing an sACE2-Fc variant in mice and NHPs,^31^ our method has a number of (theoretical) advantages: (i) HSC gene therapy might be less affected by antivector immune responses that can limit the duration of transgene expression than the rAAV-based airway transduction approach.^47^ (ii) As we have shown for factor VIII, the protein is either actively secreted from erythroid cells in the bone marrow or passively released from erythrocytes during senescence. Both mechanisms could result in efficient systemic distribution of the decoy receptor. This, in turn, could prevent viremia and infection of other organs. (iii) The insert capacity of the HDAd5/35^++^ vectors is 35 kb compared to 4.7 kb for rAAV vectors.

This would allow the accommodation of multiple effector cassettes into one HDAd5/35^++^ vector, for example, an sACE2-Ig plus a cassette for the expression of a broadly neutralizing antibody(ies). (iv) Unlike rAAV production, manufacturing of HDAd5/35^++^ vectors does not involve large-scale plasmid transfection and is technically easier and less expensive.

Our work was started early in the pandemic, before information on improved sACE2-Ig variants was available. We used the native extracellular ACE2 domain (residue 18–740)^36^ and inserted mutations that avoid potential cardiovascular effects.^37^ This decoy domain was fused to immunoglobulin elements that we used before to create secreted receptor proteins.^48^ The size of the transgene cassette inserted into an HDAd5/35^++^ vector was 12.6 kb and would therefore by too large for use in rAAV and lentivirus vectors. After *in vivo* transduction of CD46tg mice, the neutralizing activity of sACE2-Ig in serum was about 800 ng/mL and comparable with earlier studies that used 293 cells to produce rACE2-Fc protein.^36,37^ We therefore conclude that erythroid cells are capable of expressing and secreting sACE2-Ig in an active form.

Although we reached high plasma concentrations of sACE2-Ig, the activity of our construct is insufficient to completely block infection with the SARS-CoV2-surrogate or a SARS-CoV virus isolate. This became apparent in the CoV2 challenge study in K18-ACE2 mice expressing sACE2-Ig from erythroid cells. In our studies, the effect of sACE2-Ig expression on viral titers in the serum was not apparent in animals euthanized on day 2, but became pronounced at later time points, indicating sACE2-Ig acts to hinder viremic spread of *de novo* produced virus.

While the viral titers measured in lung tissue were not significantly different, consequences of lung infection such as cytokine release and lung histology were clearly improved in the test group. Despite this, all the test mice reached the endpoint and had to be euthanized by day 8. While this outcome was below our expectations, it is in line with other studies, regarding the efficacy of blocking a SARS-CoV2 challenge.^24,31^

Expression of sACE2-Ig after *in vivo* transduction of CD46tg mice with serum levels ∼1 μg/mL did not show any side effect over the time frame of the study, which was 22 weeks in primary mice and 9 weeks in secondary mice. This indicates that expression of an added protein is tolerated by erythroid cells. This is in agreement with our previous studies using factor VIII as a transgene.^12^ To test the safety of the approach in a more adequate model, we performed *in vivo* HSC transduction/selection with HDAd-sACE2-Ig in a rhesus macaque.

This is part of our larger effort to assess the *in vivo* technology for potential clinical application.^14^ It is the first study with a secreted protein. We confirmed that intravenous HDAd5/35^++^ injection of 3.2 × 10^12^ vp/kg (split into two injections) was well tolerated after appropriate cytokine prophylaxis (dexamethasone, IL-6R monoclonal antibody tocilizumab, and IL-1 blocker anakinra). Furthermore, sACE2-Ig expression from erythroid cells did not affect erythropoiesis and red blood cell functions.

In contrast to lentiviral^49^ or adeno-associated virus vectors,^50^ SB100x-mediated integration is not biased toward insertion into or near genes,^51^ leading to a lower risk of genotoxic events. This has been confirmed in our HDAd5/35^++^-based *in vivo* HSC transduction studies in mice^3,52–55^ and NHPs^14^ by genome-wide analysis of integration sites and alterations in the transcriptome. In all studies, the integration was random. No accumulation of integration sites, indicating clonal expansion, was found. In an NHP study, only a modestly altered expression of only 53 genes was detected.^14^ None of these genes was proto-oncogenic or related to cancer pathways. Overall, this indicates that, in the context of our approach and vector system, SB100x-mediated integration is safe.

In summary, our studies with HDAd-sACE2-Ig in mice and a rhesus macaque suggest that *in vivo* HSC transduction and sACE2-Ig expression in erythroid cells are well tolerated. Our *in vivo* approach significantly diminished clinical and pathologic consequences of SARS-CoV2 challenge in a mouse model. However, the blocking activity of our construct was suboptimal for complete control of a high-titer SARS-CoV2 challenge. Newer versions of sACE2-Ig will have to be used in future studies, which include ACE2 affinity-enhanced^56,57^ and optimized Fc domains,^31^ as well as species-specific constructs to avoid immune responses.

## Supplementary Material

Supplemental data

Supplemental data

Supplemental data

Supplemental data
