# Supplementary material for: In Vivo Hematopoietic Stem Cell Gene Therapy for SARS-CoV2 Infection Using a Decoy Receptor
Source: Hum Gene Ther. 2022 Apr 19;33(7-8):389–403. doi: 10.1089/hum.2021.295 (PMC9063208; doi:10.1089/hum.2021.295)
Supplement: Supplemental data [file Supp_Data.pdf]

Supplementary Methods

Reagents: G-CSF (Neupogen™) (Amgen Thousand Oaks, CA), AMD3100 (Sigma-Aldrich, St. Louis, MO) were used. O^6-BG and BCNU were from Sigma-Aldrich (St, Louis, MO)

Generation of HDAd5/35++ vectors. The DNA fragment containing the enhanced Sleeping Beauty transposon terminal inverted repeats (PT4 IRs) (sequences are based on the plasmid PT4/HB (Addgene, #108352)) flanked by FRT sites was synthesized by Genscript (Nanjing, China) and inserted into a shuttle plasmid based on the cosmid vector pWE15 (Stratagene) (pWEH-PT4). The DNA fragment containing murine IgG kappa signal peptide, HA tag and the cDNA encoding for the extracellular domain (residue 18-740) of human ACE2 [32] fused to human constant IgG(γ1) domains was synthesized by Genscript (Nanjing, China) and ligated with BstBI cleaved pBS-µLCR-ET3-mgmt [12] (pBS-µLCR-sACE2-Ig-mgmt). The two active-site histidine residues of ACE2 were mutated (H374N and H378N) to reduce the catalytic activity [33]. The 12.6kb µLCR-β-sACE2-Ig-Ef1α-mgmt transposon was released from pBS-µLCR-sACE2-Ig-mgmt by PacI digestion and inserted into the PacI site of pWEH-PT4 between the two PT4 IRs (pWEH-PT4- µLCR-sACE2-Ig-mgmt). The resulting plasmids were packaged into phages using Gigapack III plus packaging Extract (Agilent Genomics) and propagated. For the production of HDAd-sACE2-Ig-mgmt virus, pWEH-PT4-µLCR-sACE2-Ig-mgmt was linearized with FseI and rescued in 116 cells with AdNG163-5/35++, an Ad5/35++ helper vector containing chimeric fibers composed of the Ad5 fiber tail, the Ad35 fiber shaft, and the affinity-enhanced Ad35++ fiber knob.

Generation of sACE2-Ig protein. To generate the plasmid for transient expression of sACE2-Ig, the DNA fragment containing signal peptide-HA tag-sACE2-Ig was PCR amplified from the plasmid pBS-µLCR-sACE2-Ig-mgmt and inserted into pCDNA3.1(+) after the CMV promoter. The FreeStyle 293 expression system

(Life technologies) was used to express the fusion sACE2-Ig protein. Briefly, HEK293F cells were grown in suspension using FreeStyle 293 expression medium at 37°C in a humidified 8% CO_2 incubator rotating at 130rpm. 100ml culture with a density of 1 million cells per ml were transfected using FreeStyle™ MAX reagent and cultured for 3 days, the supernatant was harvested and cells were cultured in fresh medium for another 4 days for the second harvest. Proteins were purified from clarified supernatants by using Protein A resin (Genscript, cat# L00210), and then dialyzed with a buffer containing 25 mM Tris pH7.5, 200 mM NaCl, 0.2 mM $ZnCl_2$, and 10% glycerol.

Western blotting. MO7e cells were transduced with HDAd-sACE2-Ig vector at an MOI of 1500 vp/cell. Cell pellet and culture medium (secreted) were collected at 24, 48 and 72 hours post infection. Cell pellets were then subjected to sonication. The lysate from sonication or culture medium were separated by polyacrylamide gel electrophoresis and then transferred onto nitrocellulose membranes. Anti HA-tag antibody (Cell Signaling, cat# 2999) and anti-human IgG(Fc) antibody (AbD Serotec, cat# AHP1323p) were used to detect N- and C-terminal, respectively. β-actin antibodies were used as a loading control.

Measurement of VCN. Total DNA from bone marrow cells was extracted using the *Quick*-DNA Miniprep kit (Zymo Research). Viral DNA extracted from HDAd-sACE2-Ig virus was serially diluted and used for a standard curve. qPCR was conducted in triplicate using the *Power* SYBR Green PCR Master Mix on a StepOnePlus real-time PCR system. For a 10 µL reaction 9.6 ng DNA (9600 pg/6 pg/cell = ~1600 cells) was used. The following primer pairs were used: human mgmt^{P140K} forward, 5′- tgagaggcaatcctgtcaag-3′, and reverse, 5′- CAACCGGTGGCCTTCATGGG-3′.

NHP treatment:

Antibiotics: CEFTAZIDIME/TAZICET (Hospira, Inc): 150mg/kg daily, intravenous (IV), start day of surgery, continued through study as long as tether is in); FLUCONAZOLE (Northstar Rx): 50mg- flat dose per animal, IV, start on D-5 per os (PO), semil in die-once daily (SID); ACYCLOVIR (AuroMedics Pharma): 10mg/kg, IV, Start on D-5 SID

Anti-emetic/hypotension prophylaxis: Lactated Ringer Solution/LRS BOLUS: 8ml/kg, IV SID D-1 and D0, give after 2nd HDAd5/35++ infusion over 15 minutes; MAROPITANT (CERENIA) 1mg/kg, IV SID D-1 and D0, give prior to HDAd5/35++ infusions; ONDANSETRON (Hikma Pharmaceutricals): 2mg/kg, IV SID D-1 and D0, give prior to HDAd5/35++ infusions.

Necropsy: Animals were sedated and then injected IV with an overdose of pentobarbital. Blood was flushed out from the body with 5 liters of PBS using an external perfusion pump.

Immunofluorescence on spleen sections: Spleen tissues were fresh frozen in OTC medium. After sectioning at 6 µm thickness, sections were stained with FITC-conjugated anti-HA tag antibody (Abcam, cat# ab1208) and PE-conjugated anti-CD233/band 3 extracellular domain antibody (American Research Products, cat# 08-9439-4), and analyzed by fluorescence microscopy.
