# Supplementary material for: In Vivo Hematopoietic Stem Cell Gene Therapy for SARS-CoV2 Infection Using a Decoy Receptor
Source: Hum Gene Ther. 2022 Apr 19;33(7-8):389–403. doi: 10.1089/hum.2021.295 (PMC9063208; doi:10.1089/hum.2021.295)
Supplement: Supplemental data [file Supp_FigS1.pdf]

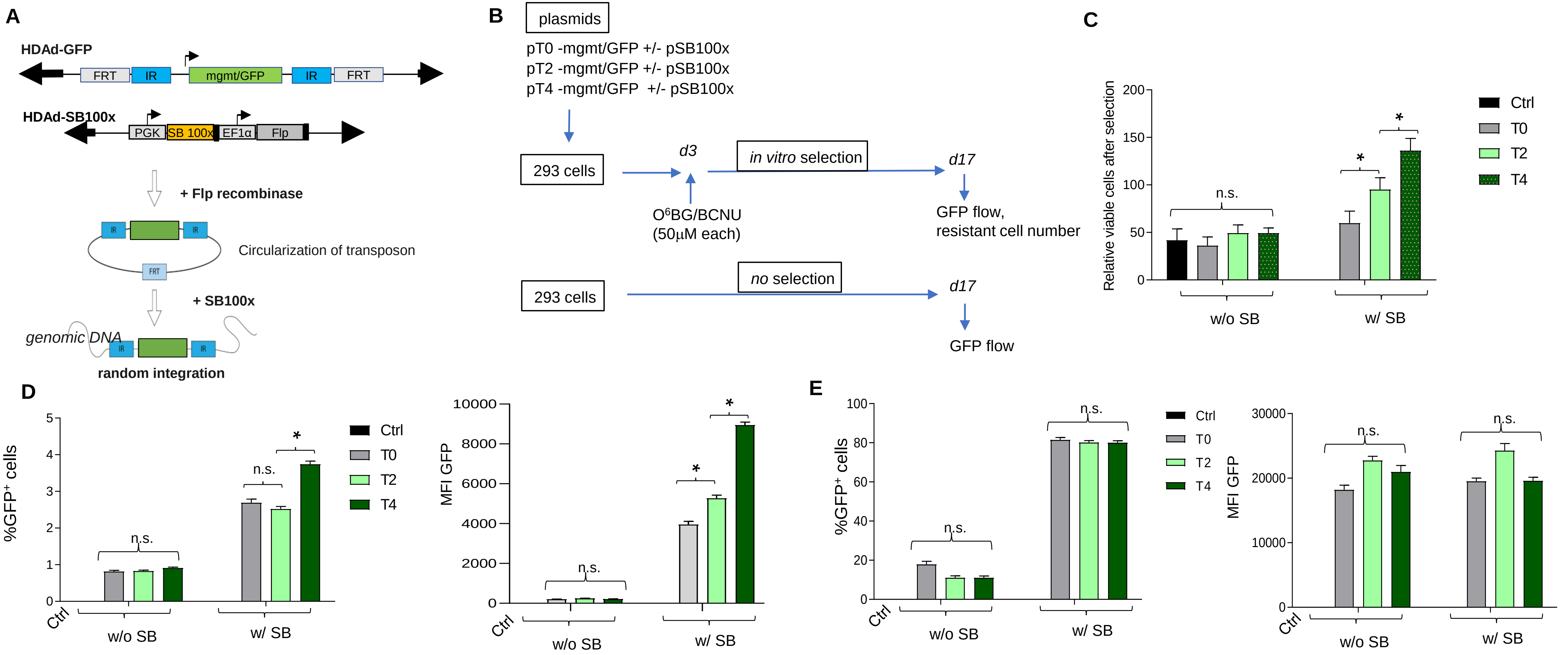

Suppl. Fig. 1. Comparison of SB100x Inverted Repeats (IR-T0, -T2, and T4). A) The SB100x system consists of an HDAd5/35++ transposon vector that carries the transgene expression cassette (e.g. mgmt/GFP), which is flanked by inverted transposon repeats (IRs) and flippase recognition target (FRT) sites. The second HDAd5/35++ vector (HDAd-SB100x) provides both Flpe recombinase and an activity-enhanced *Sleeping Beauty* transposase (SB100x) *in trans*. Upon co-infection of both vectors, Flpe mediates the circularization of the transposon through the FRT sites. SB100x then randomly integrates the transposon into the host genome through interaction with the Irs (see ref. 7). So far, we employed T0 as an IR used in all of our published vectors. T2 is an IR that is used clinically for CAR T-cell therapy (ref. 35) T4 is another version of an IR developed recently (ref.34). So far, T0, T2, and T4 have not been compared side-by-side. **B)** We constructed three plasmids containing the same GFP/mgmt^{P140K} expression cassette flanked by either T0, T2, or T4 and co-transfected them together with a SB100x expression plasmid or a control plasmid (pUC19) into 293 cells, with and without O^{6}BG/BCNU selection. (O^{6}BG/BCNU selection should kill cells where transposon (GFP/mgmt) integration did not occur). Untransfected cells and cells transfected without the SB100x-expressing plasmid served as controls. **C)** Viable cells at the end of O^{6}BG/BCNU selection. The background of surviving cells (w/o SB) is probably due to episomal plasmids. A significant difference in the number of viable cells was observed between in T0 vsT2 and T2 vsT4. *$p<0.05$. n.s.: non-significant. **D)** GFP expression without O^{6}BG/BCNU selection measured at day 17 after transfection. In the absence of SB100x, GFP expression originates from residual episomal plasmids and, as expected, no difference was observed. In the presence of SB100x, integration occurred. The percentage of GFP+ cells was comparable for T0 and T2, but significantly higher for T4. The GFP MFI reflects the GFP expression level, i.e. the number of integrated transposon copies per cell. Again, the MFI for T4 was significantly higher. **E)** GFP expression with O^{6}BG/BCNU selection. As expected, all selected cells expressed GFP to a comparable degree. Based on these data, we chose to use pT4 IRs to flank the transposon in HDAd-sACE2-Ig.
