# Supplementary material for: In Vivo Hematopoietic Stem Cell Gene Therapy for SARS-CoV2 Infection Using a Decoy Receptor
Source: Hum Gene Ther. 2022 Apr 19;33(7-8):389–403. doi: 10.1089/hum.2021.295 (PMC9063208; doi:10.1089/hum.2021.295)
Supplement: Supplemental data [file Supp_FigS2.pdf]

left IR/DR

```
     1        10        20        30        40        50        60        70        80        90        100       110       120       130
PT0  CCCTACAGTTGAAGTCGGAAGTTTACATACACTTAAGTTGGAGTCATTAAAACTCGTTTTTCAACTACTCCACAAATTTCTTGTTAACAAACAATAGTTTTGGCAAGTCAGTTAGGACATCTACTTTGTG
PT0' CTATACAGTTGAAGTCGGAAGTTTACATACACTTAAGTTGGAGTCATTAAAACTCGTTTTTCAACTACTCCACAAATTTCTTGTTAACAAACAATAGTTTTGGCAAGTCAGTTAGGACATCTACTTTGTG
PT2    CTACAGTTGAAGTCGGAAGTTTACATACACTTAAGTTGGAGTCATTAAAACTCGTTTTTCAACTACACCACAAATTTCTTGTTAACAAACAATAGTTTTGGCAAGTCAGTTAGGACATCTACTTTGTG
PT4     TACAGTTGAAGTCGGAAGTTTACATACACTTAAGTTGGAGTCATTAAAACTCGTTTTTCAACTACTCCACAAATTTCTTGTTAACAAACAATAGTTTTGGCAAGTCAGTTAGGACATCTACTTTGTG

     131     140       150       160       170       180       190       200       210       220       230       240       250       260
PT0  CATGACACAAGTCATTTTTCCAACAATTGTTTACAGACAGATTATTTCACTTATAATTCACTGTATCACAATTCCAGTGGGTCAGAAGTTTACATACACTAAGTTGACTGTGCCTTTAAACAGCTTGGAA
PT0' CATGACACAAGTCATTTTTCCAACAATTGTTTACAGACAGATTATTTCACTTATAATTCACTGTATCACAATTCCAGTGGGTCAGAAGTTTACATACACTAAGTTGACTGTGCCTTTAAACAGCTTGGAA
PT2  CATGACACAAGTCATTTTTCCAACAATTGTTTACAGACAGATTATTTCACTTATAATTCACTGTATCACAATTCCAGTGGGTCAGAAGTTTACATACACTAAGTTGACTGTGCCTTTAAACAGCTTGGAA
PT4  CATGACACAAGTCATTTTTCCAACAATTGTTTACAGACAGATTATTTCACTTATAATTCACTGTATCACAATTCCAGTGGGTCAGAAGTGTACATACACGCGCTTGACTGTGCCTTT

     261     270       280       290 294
PT0  AATTCCAGAAAATGATGTCATGGCTTTAGAAGCT
PT0' AATTCCAGAAAATGATGTCATGGCTTTAGAAGCT
PT2  AATTCCAGAAAATGATGTCATGGCTTTAGAAGCT
PT4
```

right IR/DR

```
     1        10        20        30        40        50        60        70        80        90        100       110       120       130
PT0  AGCTTGTGGAAGGCTACTCGAAATGTTTGACCCAAGTTAAACAATTTAAAGGCAATGCTACCAAATACTAATTGAGTGTATGTTAACTTCTGACCCACTGGGAATGTGATGAAAGAAATAAAAGCTGAAA
PT0' AGCTTGTGGAAGGCTACTCGAAATGTTTGACCCAAGTTAAACAATTTAAAGGCAATGCTACCAAATACTAATTGAGTGTATGTTAACTTCTGACCCACTGGGAATGTGATGAAAGAAATAAAAGCTGAAA
PT2  AGCTTGTGGAAGGCTACTCGAAATGTTTGACCCAAGTTAAACAATTTAAAGGCAATGCTACCAAATACTAATTGAGTGTATGTAAACTTCTGACCCACTGGGAATGTGATGAAAGAAATAAAAGCTGAAA
PT4                                      TTAAACAATTTAAAGGCAATGCTACCAAATACTAAGCGCGTGTATGTACACTTCTGACCCACTGGGAATGTGATGAAAGAAATAAAAGCTGAAA

     131     140       150       160       170       180       190       200       210       220       230       240       250       260
PT0  TGAATCATTCTCTCTACTATTATTCTGATATTTCACATTCTTAAAATAAAGTGGTGATCCTAACTGACCTTAAGACAGGGAATCTTTACTCGGATTAAATGTCAGGAATTGTGAAAAAGTGAGTTTAAAT
PT0' TGAATCATTCTCTCTACTATTATTCTGATATTTCACATTCTTAAAATAAAGTGGTGATCCTAACTGACCTTAAGACAGGGAATCTTTACTCGGATTAAATGTCAGGAATTGTGAAAAAGTGAGTTTAAAT
PT2  TGAATCATTCTCTCTACTATTATTCTGATATTTCACATTCTTAAAATAAAGTGGTGATCCTAACTGACCT-AAGACAGGGAATTTTTACTAGGATTAAATGTCAGGAATTGTGAAAAAGTGAGTTTAAAT
PT4  TGAATCATTCTCTCTACTATTATTCTGATATTTCACATTCTTAAAATAAAGTGGTGATCCTAACTGACCTTAAGACAGGGAATCTTTACTCGGATTAAATGTCAGGAATTGTGAAAAAGTGAGTTTAAAT

     261     270       280       290       300       310   315
PT0  GTATTTGGCTAAGGTGTATGTAAACTTCCGACTTCAACTGTAGGGGATCCTCTAG
PT0' GTATTTGGCTAAGGTGTATGTAAACTTCCGACTTCAACTGTAGG
PT2  GTATTTGGCTAAGGTGTATGTAAACTTCCGACTTCAACTGTATAG
PT4  GTATTTGGCTAAGGTGTATGTAAACTTCCGACTTCAACTGTA
```

Suppl.Fig.2. Nucleotide alignment of left and right IR for the previously published T0 version, the T0 version used so far in our HDAd vectors, the T2 version, and the T4 version.
