# Supplementary material for: In Vivo Hematopoietic Stem Cell Gene Therapy for SARS-CoV2 Infection Using a Decoy Receptor
Source: Hum Gene Ther. 2022 Apr 19;33(7-8):389–403. doi: 10.1089/hum.2021.295 (PMC9063208; doi:10.1089/hum.2021.295)
Supplement: Supplemental data [file Supp_FigS3.pdf]

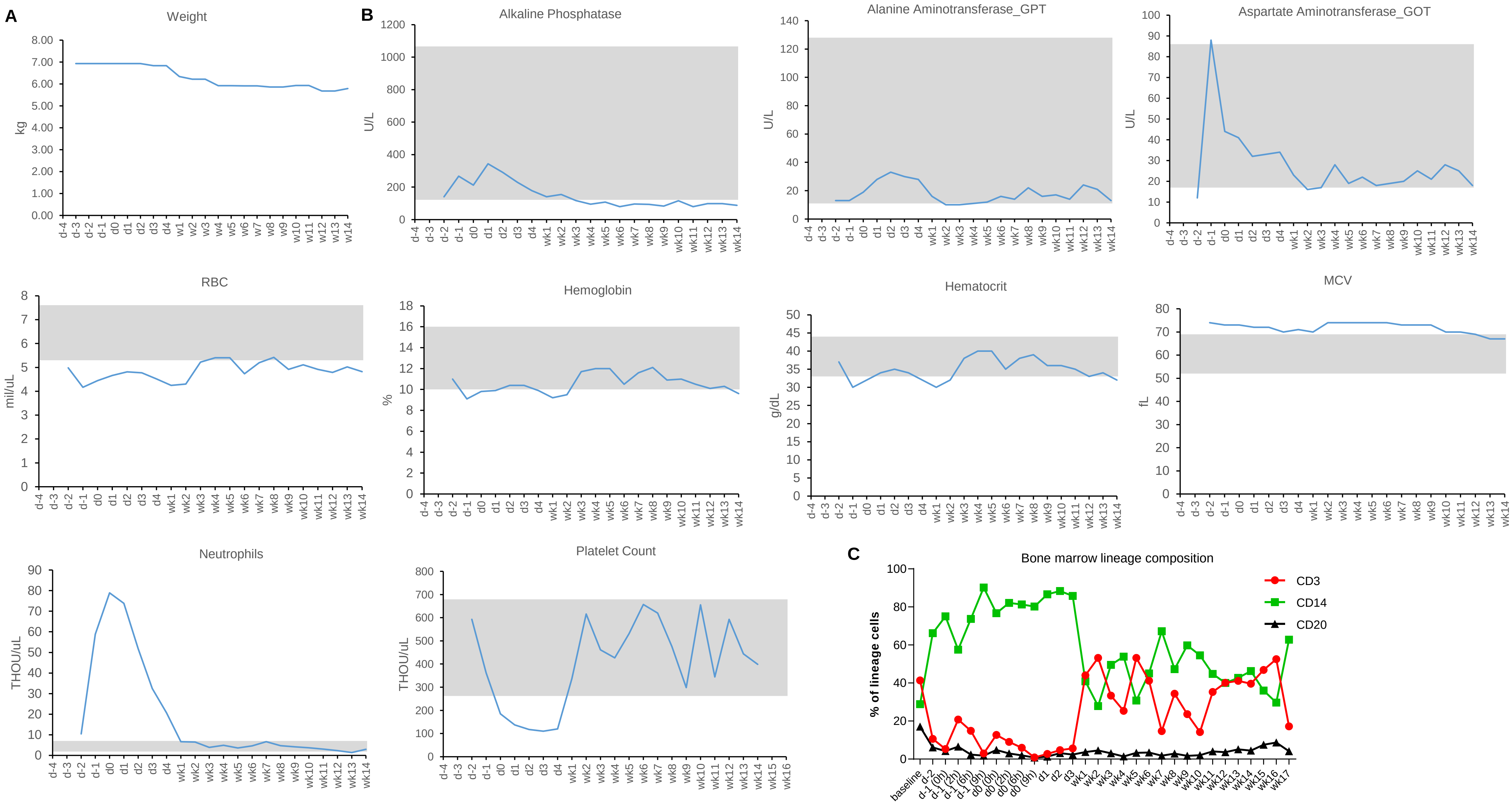

Suppl. Fig. 3. Effects of *in vivo* HSC transduction with HDAd-sACE2-Ig/HDAd-SB on weight (A), blood parameters (B) and bone marrow cell composition (C). The grey shaded area indicates the normal range of a given parameter for rhesus macaques.
